# Age-related changes of whole-brain dynamics in spontaneous neuronal coactivations

**DOI:** 10.1038/s41598-022-16125-2

**Published:** 2022-07-15

**Authors:** Guofa Shou, Han Yuan, Yoon-Hee Cha, John A. Sweeney, Lei Ding

**Affiliations:** 1 Stephenson School of Biomedical Engineering, University of Oklahoma, Norman, USA; 2 Institute for Biomedical Engineering, Science, and Technology, University of Oklahoma, Norman, USA; 3 Department of Neurology, University of Minnesota, Minneapolis, MN USA; 4 Department of Psychiatry, University of Cincinnati, Cincinnati, OH USA; 5 University of Oklahoma, 173 Felgar St., Gallogly Hall, Room 101, Norman, OK 73019 USA

**Keywords:** Biomedical engineering, Neural ageing

## Abstract

Human brains experience whole-brain anatomic and functional changes throughout the lifespan. Age-related whole-brain network changes have been studied with functional magnetic resonance imaging (fMRI) to determine their low-frequency spatial and temporal characteristics. However, little is known about age-related changes in whole-brain fast dynamics at the scale of neuronal events. The present study investigated age-related whole-brain dynamics in resting-state electroencephalography (EEG) signals from 73 healthy participants from 6 to 65 years old via characterizing transient neuronal coactivations at a resolution of tens of milliseconds. These uncovered transient patterns suggest fluctuating brain states at different energy levels of global activations. Our results indicate that with increasing age, shorter lifetimes and more occurrences were observed in the brain states that show the global high activations and more consecutive visits to the global highest-activation brain state. There were also reduced transitional steps during consecutive visits to the global lowest-activation brain state. These age-related effects suggest reduced stability and increased fluctuations when visiting high-energy brain states and with a bias toward staying low-energy brain states. These age-related whole-brain dynamics changes are further supported by changes observed in classic alpha and beta power, suggesting its promising applications in examining the effect of normal healthy brain aging, brain development, and brain disease.

## Introduction

Human brains experience significant changes across the lifespan. At different life stages, i.e., infant, toddler, adolescent, adulthood, and senescence, changes in normal development, maturation, and degeneration occur in both brain anatomy and function^1–3^. Meanwhile, neuropsychiatric disorders of age-specific incidences have been reported to be significantly correlated to abnormalities in brain anatomy and/or function^4–6^. Therefore, it is critical to understand changes in normal aging human brains, which is the basis for understanding and treating brain diseases at different ages. Numerous studies have investigated age-related anatomic and functional changes in human brains utilizing various neuroimaging modalities^2,7–10^. Structural magnetic resonance imaging (sMRI)^11^ has shown age-related changes in white and gray matters in which white matter volume follows an inverted U-shape curve with a maturation peak around mid-life whereas gray matter volume continuously decreases throughout the lifespan^9,12^. Correspondingly, domain-specific age-related functional changes (e.g., cognition and memory) have also been extensively studied using functional MRI (fMRI)^7,10,13^, positron emission tomography (PET)^14,15^, electroencephalography (EEG)^2,16–18^, and magnetoencephalography (MEG)^8^.

While regional and whole-brain changes have been reported under both task and resting conditions, regional changes are more reported in tasks, suggesting functional domain-specific age-related changes. A meta-analysis of fMRI and PET studies revealed age-related deficits in working and episodic memory abilities that are linked to two distinct regional changes within the prefrontal cortex (PFC)^7^, i.e., functional differentiation in bilateral ventral PFC and functional deficits in right dorsal and anterior PFC. A fMRI study found age-related linear hemodynamic signal decreases in task-activated areas but non-linear increases in task-unrelated areas during episodic memory tasks^10^. An EEG study reported increased beta oscillations indicating greater GABAergic inhibitory activity within motor cortices of older than younger adults^19^. On the contrary, whole-brain age-related changes have mainly been reported in resting-state neuroimaging studies^8,13,17,18,20–22^. A resting-state fMRI study reported weakened within-network functional connectivity (FC), reduced system segregation, and lowered local efficiency in all primary sensory and cognitive networks^13^. Resting-state MEG studies have shown age-related spectral power decreases in low frequencies (e.g., delta) over most sensors covering the whole head^8,23^. Spectrally-resolved resting-state EEG topographic maps have revealed microstates from the delta to gamma bands showing significant age-related global differences in their spatial maps^20^. In these investigations, band-specific EEG/MEG power data^8,17^ and fMRI amplitude data^10,17^ were commonly used to evaluate regional changes (i.e., intra-region measures), while FC measures computed from either neurophysiological^16^ or hemodynamic signals^13,24,25^ evaluating dependence, interaction, and integration between regions are mainly used for cross-region and whole-brain changes (i.e., inter-region measures). It is worth noting that age-related whole-brain changes have been observed in studies using both intra-region^1,8,17^ and inter-region measures^8,13^.

As the studies discussed above have focused on examining spatial patterns using both intra- and inter-region measures, recent studies have paid increasing attention to age-related temporal dynamics. Complexity and variability of region-specific signals are two widely used measures in assessing age-related changes of temporal dynamics^1,17,26^. A pattern of increase in early life but decrease in late life of complexity has been reported across the lifespan from 7 to 84 years^1^. Decreased variability from younger adults (~ 25 years) to older adults (~ 67 years) in both EEG and fMRI data have also been reported^17^. For inter-region changes, dynamic measures often focus on transient brain states^11,27^. Time-varying FC dynamics at the scale of whole brain have been used to define multiple recurring brain states that could be reliably detected and have shown age-related changes in their temporal characteristics^3,11,21,27–30^, e.g., occurrence, lifetime, and interaction. A fMRI study has reported that older adults spend much more time than young adults in a brain state characterized by weak FC throughout the brain and less time in a brain state showing strong FC within both sensory-motor and cognitive control networks^30^. Based on the measure of whole-brain phase synchronizations in BOLD signals^21^, the older adults exhibit reduced ability to access a so-called rich-club brain state (therefore lowered occurrence)^31^. In EEG, age-related changes have also been reported in the transient brain states known as microstates^20,32^, where a microstate related to cognitive processes is less visited and with reduced lifetimes. Initial evidence has further indicated that measures of brain dynamics have better sensitivity on age-related effects than measures of spatial patterns^20^, as well as in revealing disease-related changes^33^.

The present study examined age-related changes in whole-brain dynamics in healthy individuals between 6 to 65 years old by studying multiple cortical co-activation patterns (CAPs). The analysis of CAPs characterizes whole-brain dynamics with a set of transient brain states at the single-timeframe resolution^34,35^, which advances the definition of brain states from scalp-based field EEG measurements, e.g., microstates^20^, to electrical currents on anatomic structures of direct neuronal relevance. The high temporal resolution of EEG and the nature of framewise analysis in obtaining CAPs provided the capability to study much faster dynamics (i.e., up to 100 Hz) at the whole-brain scale than fMRI (< 0.1 Hz). For comparison, we also examined age-related changes using well-established static regional measures, i.e., band-specific spectral powers^2,8,16^ at the whole-brain scale^2,8,16^. We hypothesized that lifespan age-related whole-brain changes could be detected in CAPs. We further hypothesized that the lifespan process of functional brain changes should be reflected in both spatial and dynamic domains and therefore a potential link between CAP measures and spectral power measures showing whole-brain changes. Finally, given the possible confound of gender on the identification of age-related changes, we replicated the findings from the data of female-only participants.

## Results

### Whole-brain dynamic CAP measures indicating age-dependent effects

**Figure 1:**
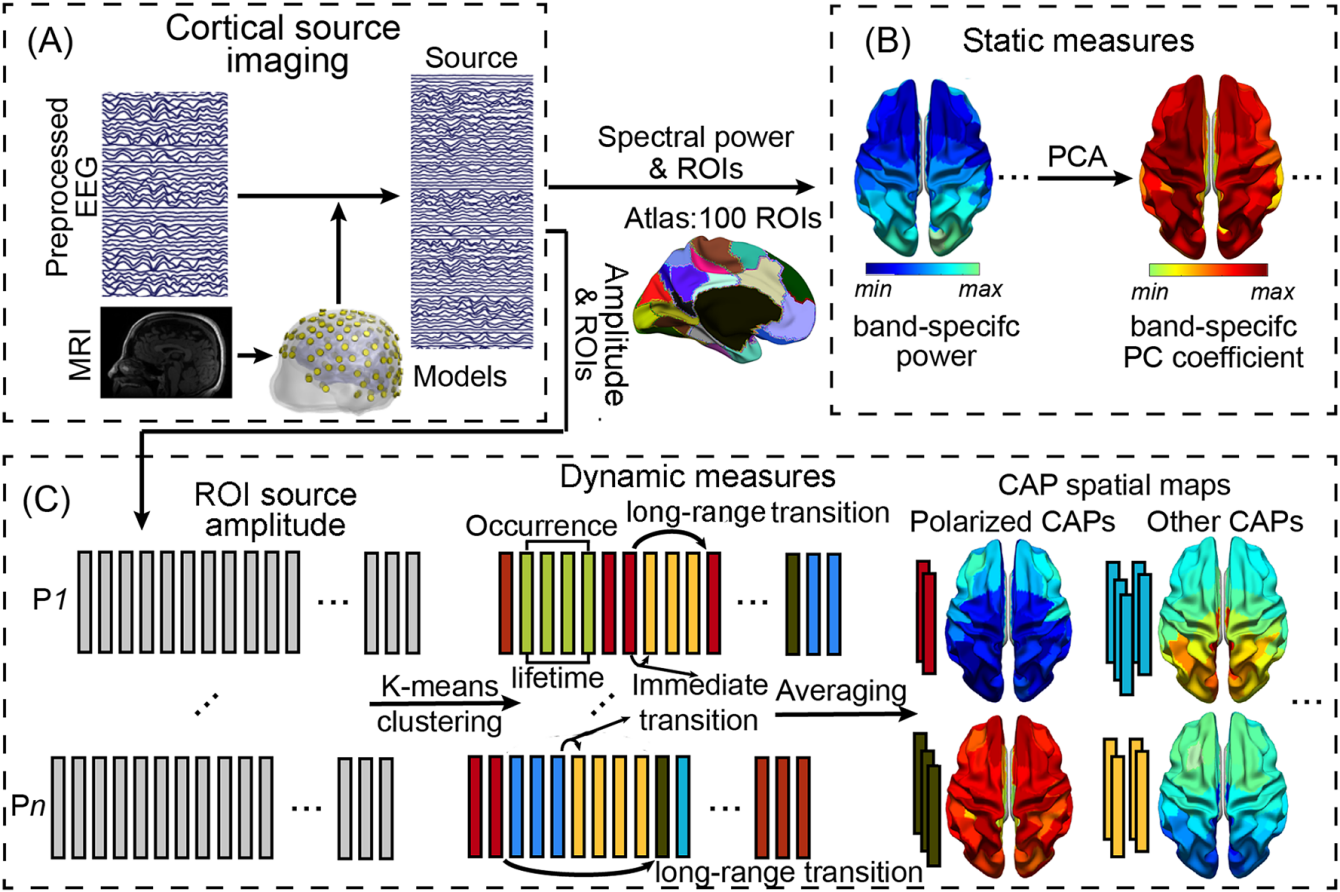
Schematic illustration of the method. (**A**) Cortical source imaging in reconstructing cortical sources from preprocessed EEG signals using individual volume conduction models from MRI; (**B**) Static measures of band-specific powers over 100 ROIs based on an atlas for the whole cortex and PCA-based whole-brain band-specific powers; (**C**) Dynamic measures defined on CAPs (identified via a k-means clustering on source envelop data at the resolution of single timeframes): the occurrence and lifetime of individual CAPs, temporal metrics involving immediate transitions from one CAP to another, and long-range transitions between two polarized CAPs (see “Methods”).

**Figure 2:**
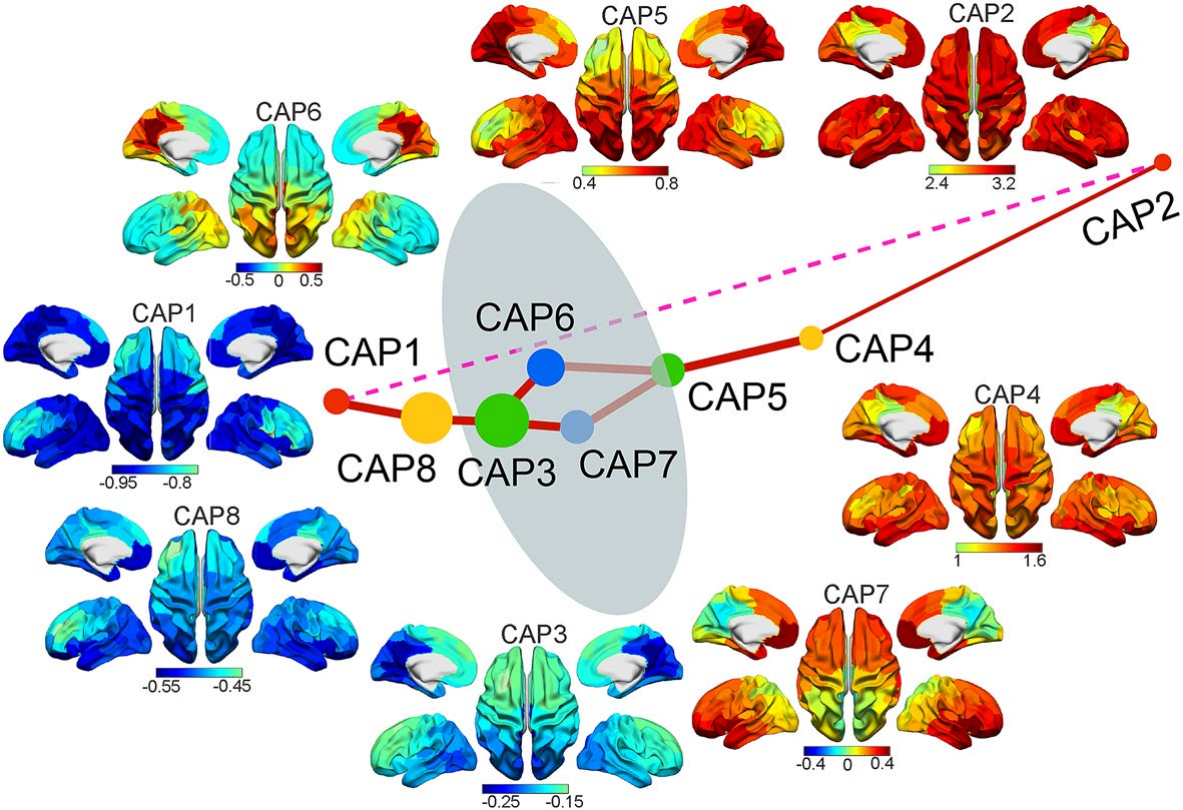
Spatial maps (z-score values) of a set of eight CAPs with spatially structured brain-wide patterns from resting EEG data and the distance map (via L1-norm distance) between these CAPs projected onto a 3D space. Each solid sphere in the distance map represents a CAP and its size is coded with the occurrence rate of the CAP. The same color of two spheres is coded for anti-correlated CAP pairs. The thicknesses of solid red lines are coded for high immediate transition probabilities thresholded at top 25% (see Supplementary Fig. 1) or the highest transition probability involving CAP2 (all transition probabilities involving CAP2 lower than the threshold). The dash pink line denotes the lowest transition probability between two polarized CAPs, i.e., the CAP1 and CAP2.

After mapping high-density resting-state scalp EEG data from total 73 participants (age 6–65 years) onto individual cortical surfaces (Fig. 1A), the clustering analysis based on single timeframe cortical tomographic current source data (Fig. 1C) led to the definition of a set of eight CAPs indicating distinct transient brain states (see “Methods” for details). Figure 2 shows their spatial maps and the high-dimensional spatial distances between these maps illustrated in 3D space (see “Methods” for its definition). These eight CAPs could be categorized into three groups (Table 1): CAPs 2, 4, and 5 having their whole-brain activations all above the mean levels (all cortical source time courses were normalized to z-scores), termed the “global high-activation group”; CAPs 1, 3, and 8 have their whole-brain activations all below the mean levels, termed the “global low-activation group”; and CAPs 6 and 7 having brain activations both above and below the corresponding mean levels over different spatial locations, termed the “non-global activation group.” Two CAPs (i.e., CAPs 1 and 2) had the highest global activation and the lowest global activation, respectively. The 3D distance plot (Fig. 2) indicates that the global high-activation CAPs are distant from the global low-activation CAPs, while the non-global CAPs are in the middle between the two global groups. In particular, the distance between CAPs 1 and 2 is the furthest at two polarized ends of the distance plot. Spatial-wise, all CAPs show bilateral symmetric patterns over the left and right hemispheres and anti-correlated pairs. Beyond two polarized CAPs, other six CAPs show high negative spatial correlations (*r* = − 0.92 for CAPs 3 and 5, − 0.98 for CAPs 6 and 7, and − 0.89 for CAPs 4 and 8). It is noted that the magnitudes of the global high-activation CAPs are in general higher than those of the global low-activation CAPs (e.g., the z-score range for CAP 2 is [2.4, 3.2] while the range for its correspondence CAP 1 is [− 0.95, − 0.8]).

**Table 1:** CAP groups defined based on spatial patterns and the summary of major age-related CAP temporal patterns from both all data and female-only data. *Not survived with FDR or Bonferroni correction (*p* < 0.05 before correction); #: approaching significance (*p* = 0.058) before FDR correction.

	CAP categories		CAP indices in all data	CAP indices in female-only data
Spatial patterns	Polarized CAPs (i.e., the lowest and highest global activations)		1, 2	6, 7
	Global high-activations		2, 4, 5	2, 4, 7
	Global low-activations		1, 3, 8	3, 5, 6
	Non-global		6, 7	1, 8
	Anti-correlated pairs		1–2, 3–5, 4–8, 6–7	1–8, 2–5, 3–4, 6–7
Temporal patterns of age-related effects (at least *p* < 0.05 after correction)	Single CAP	Occurrence (↑)	2	4
		Lifetime (↓)	2*, 4*, 5, 7*	2, 4, 7
	Transition of two polarized CAPs	Occurrence (↑)	2 → 2	6 → 6*, 7 → 7^#^
		Number of non-polarized CAPs visited	1 → 1 (↓), 2 → 2 (↑)	6 → 6 (↓), 7 → 7 (↑)

**Figure 3:**
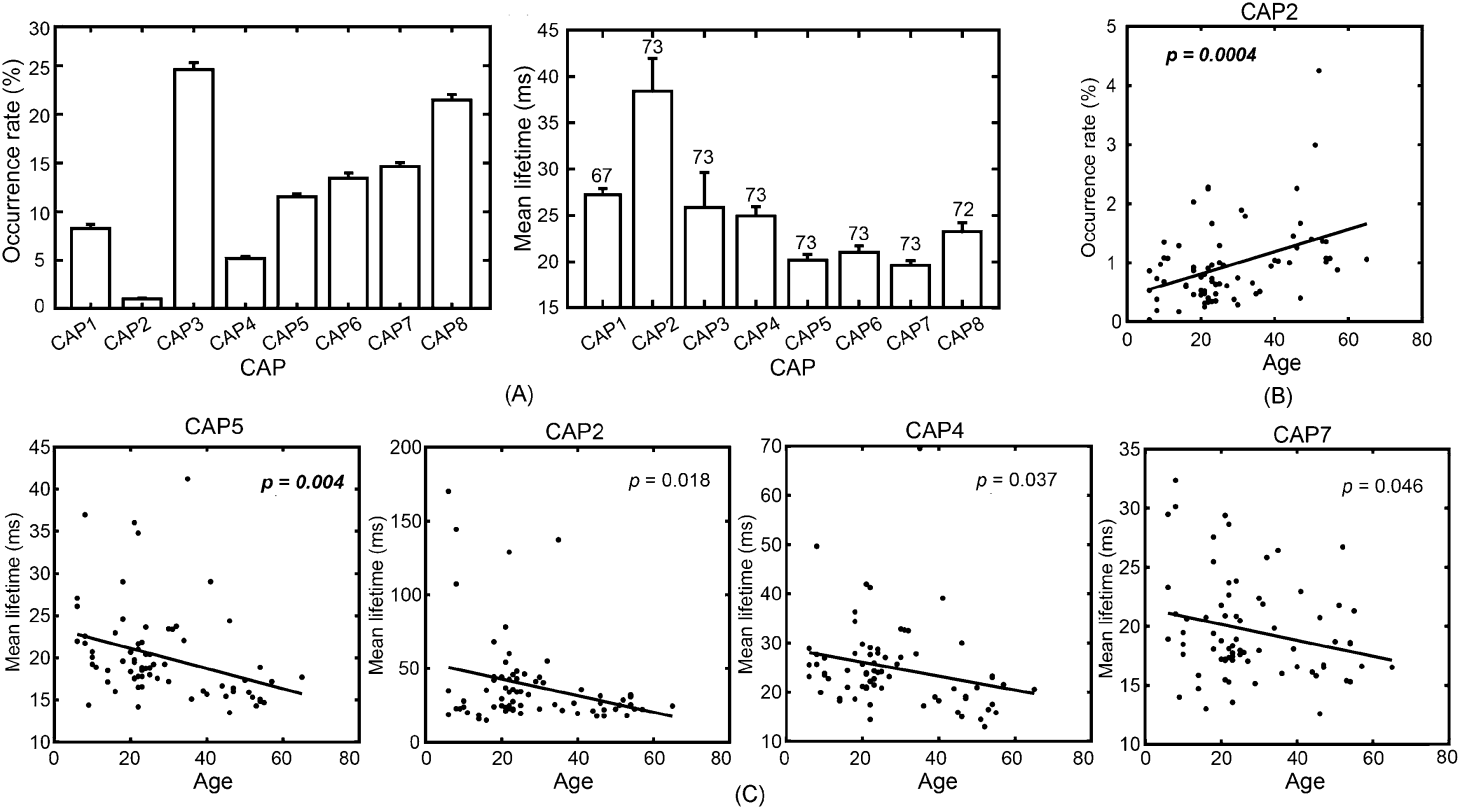
CAP-based temporal measures and their age-related patterns. (**A**) CAP occurrence rates and mean lifetimes. Numbers on each bar in mean lifetime indicate the number of participants (the total is 73) detected for a corresponding CAP. (**B**) Age-related occurrence rate changes of CAP2. (**C**) Age-related mean lifetime changes of CAPs 2, 4, 5, and 7. The lines in (**B**) and (**C**) are the linear regression models, whose *p* values are listed in the panels (bold italic fonts: survived with Bonferroni correction).

All CAPs could be reliably detected in almost all participants, i.e., six CAPs detected in all participants, with CAP1 detected in 67 participants and CAP8 detected in 72 participants out of 73 total participants. In general, both the global high-/low-activation CAPs had relatively lower occurrence rates and longer mean lifetime than the non-global CAPs while these patterns were especially outstanding for two polarized CAPs (Fig. 3A). Regarding the immediate transition data (coded in the thickness of lines connecting CAPs in Fig. 2. See actual data in Supplemental Fig. 1), the high transition probabilities were usually observed within the global high-/low-activation group, while the transitions between two global groups were usually relayed via the non-global CAPs. The close-to-zero transitions (1e−6% and 0% from CAP1 to CAP2 and vice versa, respectively) were observed between two polarized CAPs.

**Figure 4:**
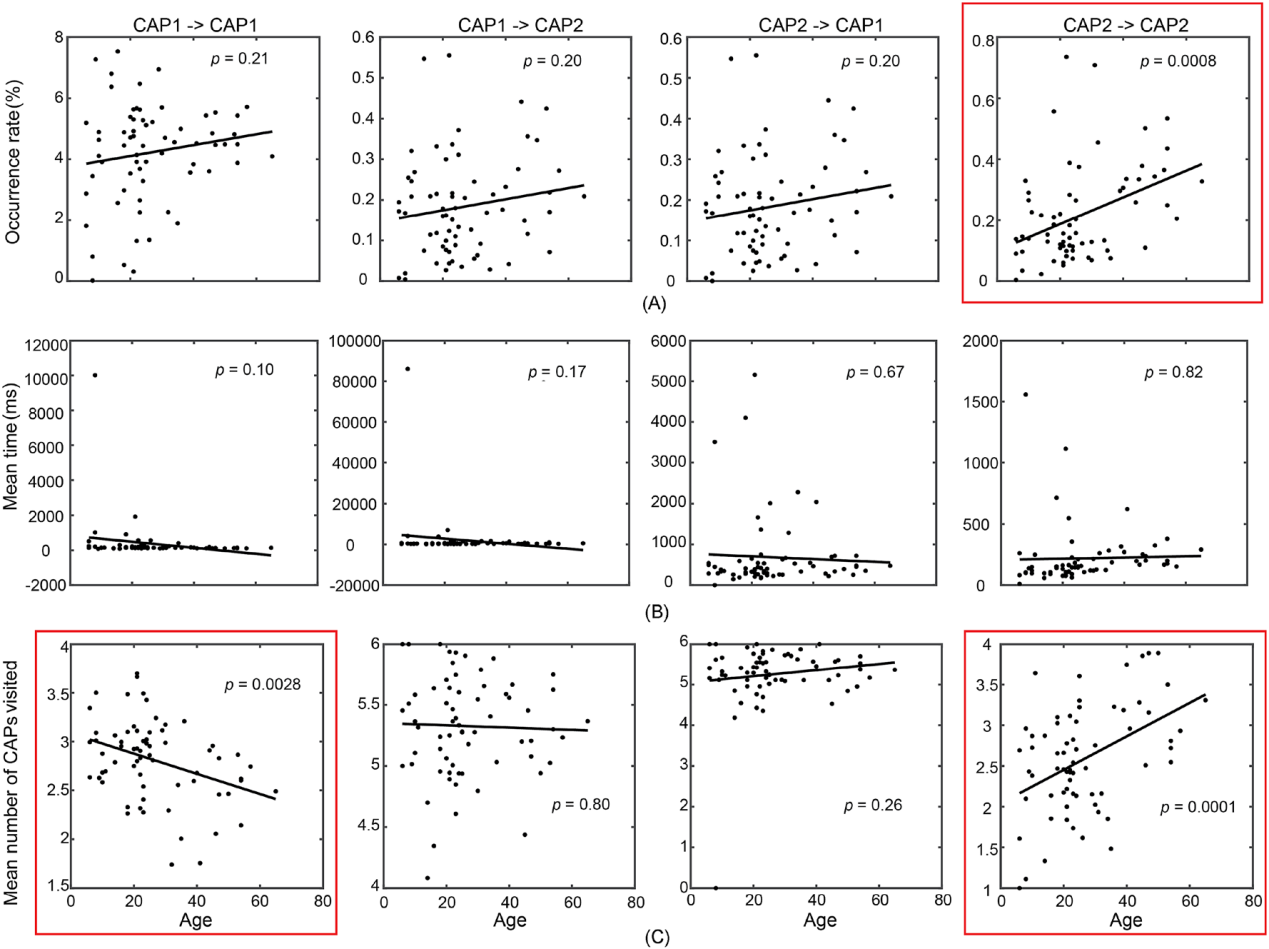
Age-related changes on measures for long-range transitions involving two polarized CAPs: (**A**) Occurrence rate; (**B**) Mean duration time; (**C**) Mean numeric counts of other CAPs visited. The line in each panel denotes the liner regression model, whose *p* values are listed in the panels (red frames: survived with Bonferroni correction).

Age-related whole-brain changes were then examined on these temporal and transitional measures of individual CAPs (see major findings in Table 1). The unique spatial, temporal, and transitional properties of the two polarized CAPs motivated the investigation of the effect of age on long-range transitions between the CAPs, which led to four types of transitions: CAP1 → CAP1, CAP1 → CAP2, CAP2 → CAP1, and CAP2 → CAP2 and three measures on these long-range transitions: occurrence rate, lifetime, and numeric counts of other brain states visited during the transition (see “Methods” for details). For the temporal measures only concerning individual CAPs, the regression analyses revealed age-related changes on the occurrence rate and mean lifetime of the CAPs mainly from the global high-activation group (Fig. 3B,C). CAP2 occurred significantly more frequently as age increased (*p* < 0.0005, Bonferroni correction, Fig. 3B). In terms of the mean lifetime (Fig. 3C), CAP5 showed a significantly decreasing trend as age increased (*p* < 0.001, Bonferroni correction), and three CAPs, i.e., 2, 4, and 7 (the only non-global high-activation CAP) showed decreasing trends as age increased (*p* < 0.05, not survived with correction). For the three long-range transitional measures, a significant increase of the occurrence rate with age was observed in CAP2 → CAP2 (*p* < 0.001, Bonferroni correction, Fig. 4A). No significant effect was detected in the mean duration of all four types of transitions (Fig. 4B). Significant effects of age on the measure of the numeric counts of other brain states visited (*p* < 0.005, Bonferroni correction, Fig. 4C) were detected in both CAP1 → CAP1 and CAP2 → CAP2, but in different directions, i.e., a significant decreasing effect in CAP1 → CAP1 and a significant increasing effect in CAP2 → CAP2.

### Static band-specific spectral power measures suggesting age-dependent effects

**Figure 5:**
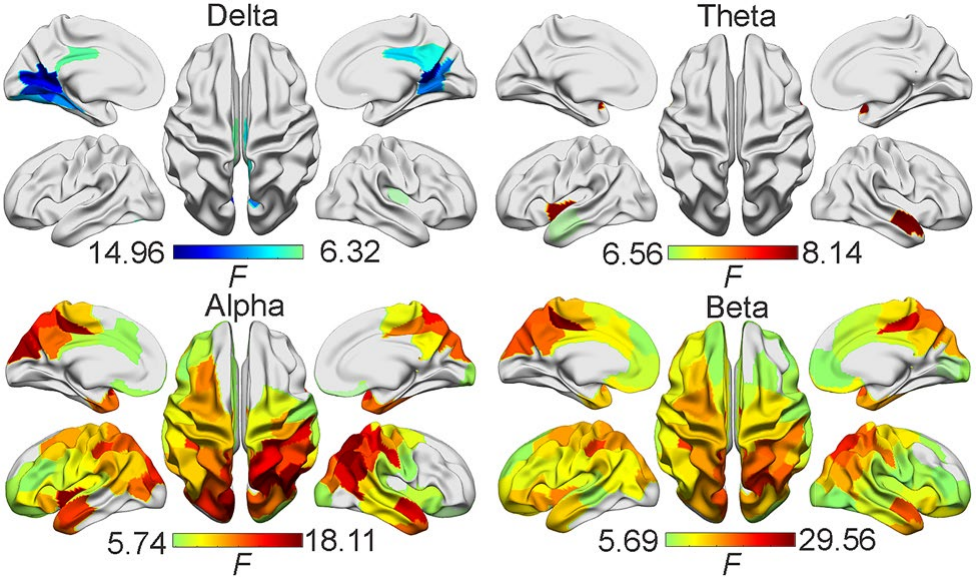
Spatial maps of significant age-related band-specific power changes with FDR correction from 4 frequency bands and 100 parcellations. Blue: decreases as age increases; Red: increases as age increases.

Figure 5 shows the detected brain regions (total 100 regions-of-interest, ROIs) over the neocortex (Fig. 1A,B and see “Methods”) with their spectral powers significantly correlating with age (*p* < 0.05, false discovery rate, FDR, correction^36^) at four different bands and 100 parcellations. It is noted that only the delta band had reduced power, and the other three bands (i.e., theta, alpha, and beta bands) had increased power with age. Moreover, in their spatial patterns, broad and almost whole-brain age-related power changes have been observed in both alpha and beta bands. Meanwhile, age-related power changes in the delta and theta bands were more regional, i.e., bilateral precuneus and the primary visual areas (V1) for the delta band and bilateral temporal cortex for the theta band.

**Figure 6:**
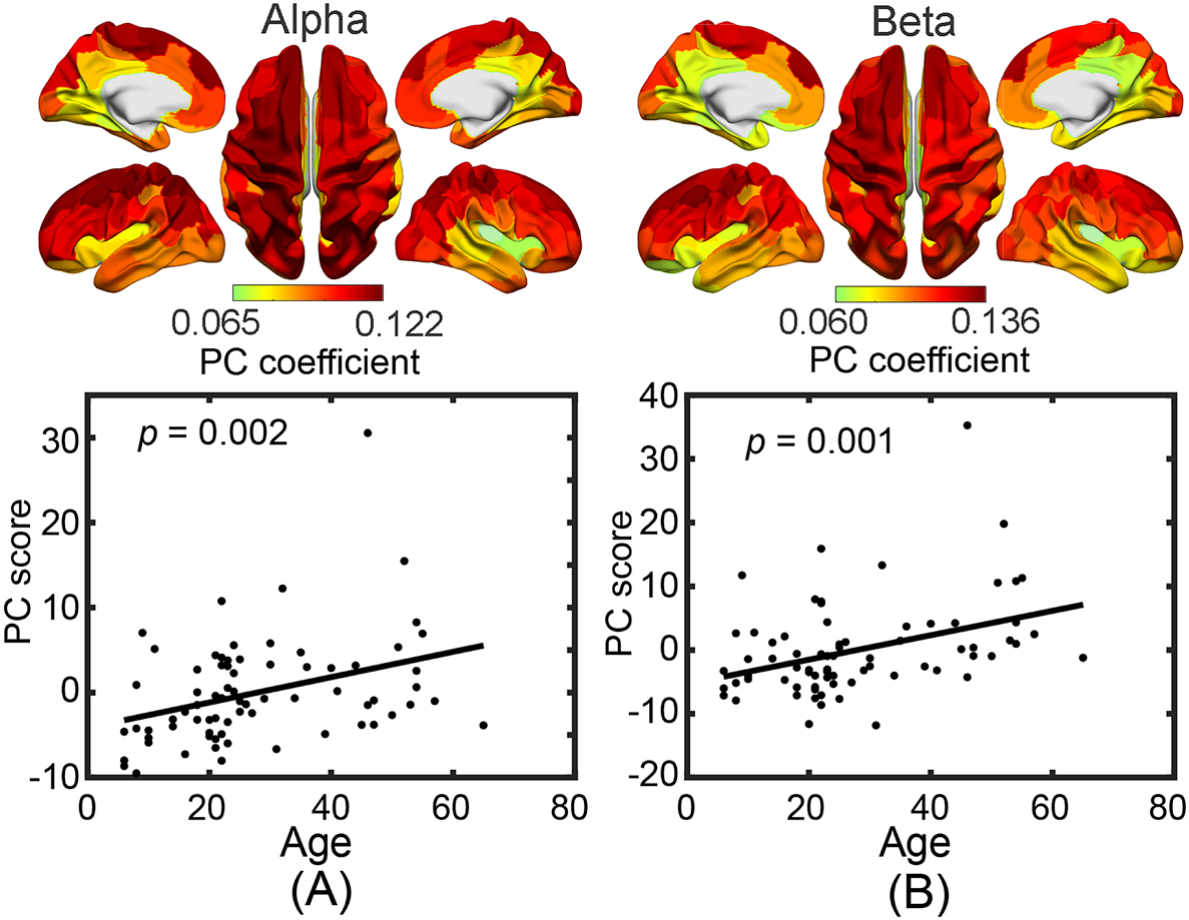
PCA-based age-related band-specific spectral power changes at the whole-brain scale. (**A**) Spatial map of PC coefficients (top) and PC scores (bottom) in the alpha band; (**B**) Spatial map of PC coefficients (top) and PC scores (bottom) in the beta band. The lines denote the linear regression models for PC scores, whose *p* values are listed (survived with Bonferroni correction).

Using principal component analysis (PCA) derived whole-brain spectral power measures (see “Methods”), the first PCs from the delta, theta, alpha, and beta bands explained variances at 74.7%, 78.5%, 76.9%, and 83.4%, respectively. The PCs showing significant age-related changes were those from the alpha and beta bands (Fig. 6), which both showed significant increasing trends as age increased (*p* < 0.05, Bonferroni correction). Their PC scores indicated that these age-related changes were widely distributed across the whole brain (the top panels in Fig. 6A,B).

### Relationship between age-related static and dynamic whole-brain measures

**Figure 7:**
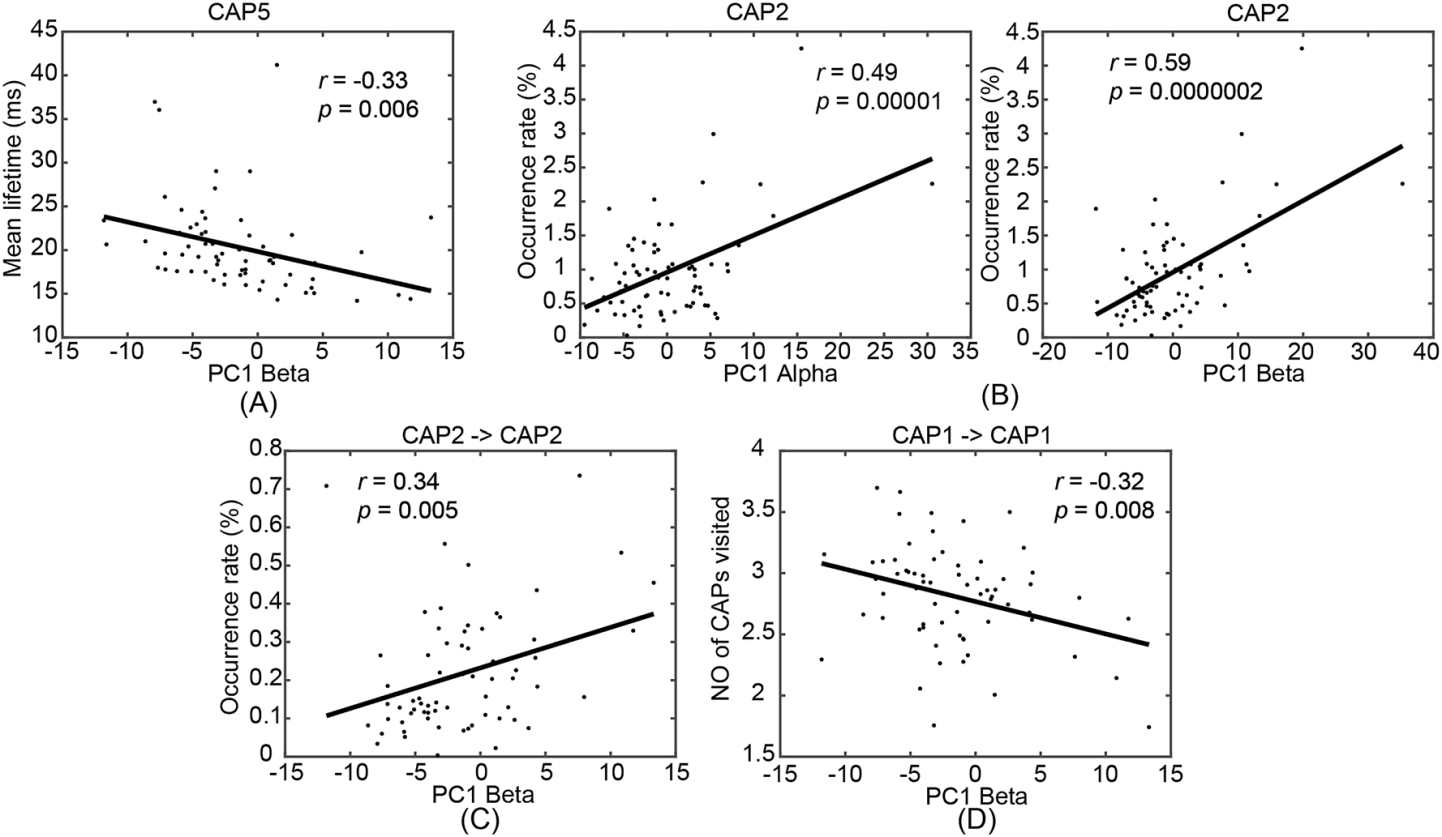
Significant correlations between age-related PCA-based static power measures and CAP-based dynamic measures (*p* < 0.01, FDR corrected). (**A**) Beta-band PC scores vs. CAP5 lifetimes; (**B**) Alpha- and beta-band PC scores vs. CAP2 occurrence rates; (**C**) Beta-band PC scores vs. CAP2 → CAP2 occurrence rates; (**D**) Beta-band PC scores vs. numeric counts of other CAPs visited in CAP1 → CAP1. The line in each panel denotes the linear regression model, while *p* and *r* values obtained from correlational analysis are listed.

It is of interest that the dominant subset of static spectral power measures indicated whole-brain age-related effects, which were spatially in line with whole-brain dynamic measures. Furthermore, the direction of these spectral power changes (i.e., increase as age increases) were consistent with increased occurrence rates of the global highest-activation brain states (i.e., CAP2) that lead to more energy consumptions. To investigate their potential dependence, correlational analyses were performed on ten pairs of one of two static spectral power measures (i.e., the first PCs in alpha and beta bands) and one of five dynamic CAP measures (i.e., occurrence rate of CAP2, lifetime of CAP5, occurrence rate of CAP2 → CAP2, numbers of other CAPs visited during CAP1 → CAP1 and CAP2 → CAP2), both showing significant age-related whole-brain effects. Five out of these ten pairs suggested significant correlations (*p* < 0.01, FDR correction, Fig. 7). The alpha band PC was only significantly correlated with one dynamic metric, i.e., occurrence rate of CAP2, which was positive (Fig. 7B). Meanwhile, the beta band PC was significantly correlated with four dynamic metrics, i.e., a positive correlation with the occurrence rate of CAP2 (Fig. 7B), a negative correlation with the lifetime of CAP5 (Fig. 7A), a positive correlation with the occurrent rate of CAP2 → CAP2 (Fig. 7C), and a negative correlation with the number of other CAPs visited during CAP1 → CAP1 (Fig. 7D).

### Reproducibility on data from female-only participants

**Figure 8:**
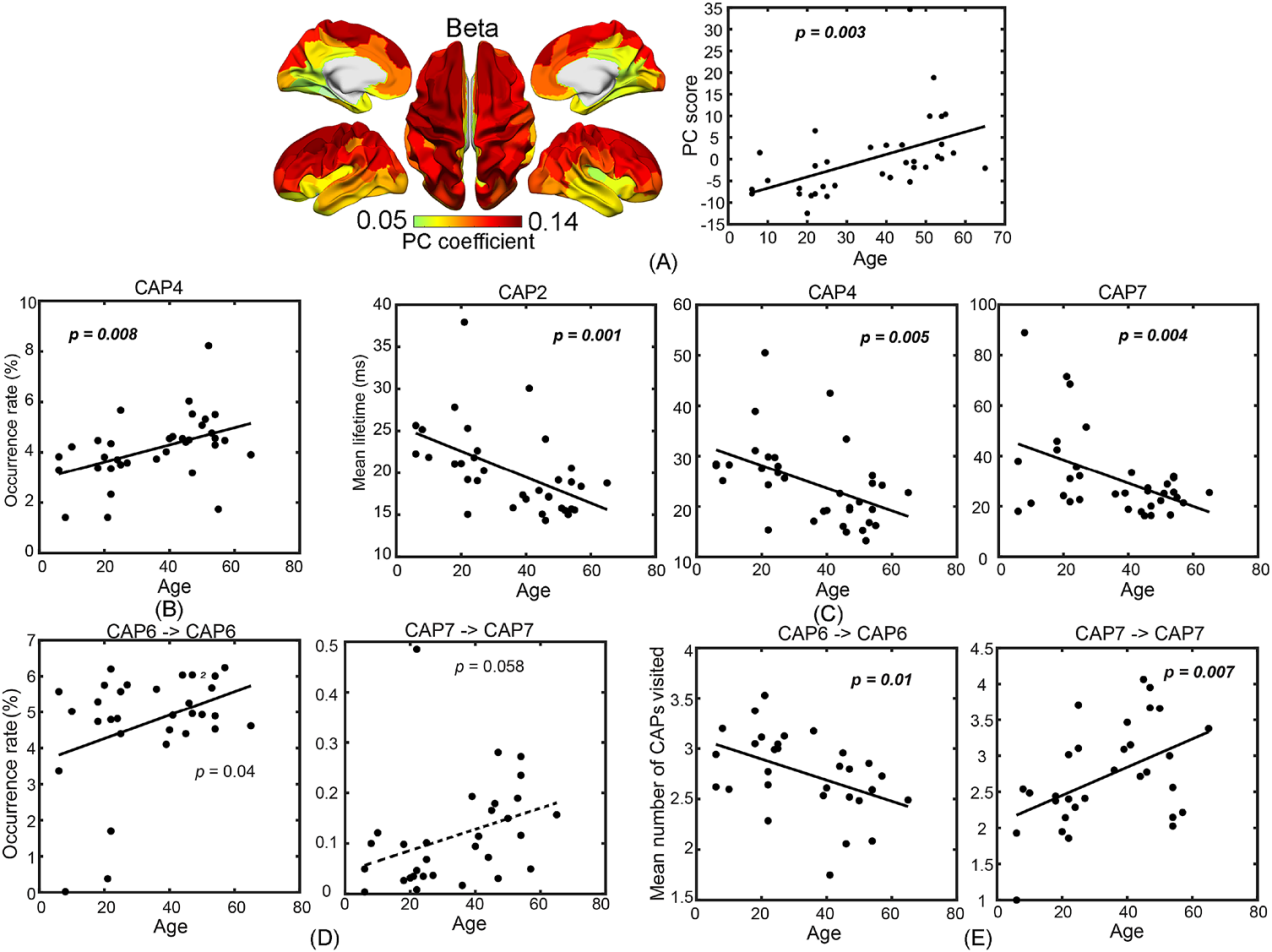
Static and dynamic measures reproduced in female-only data show significant age-related changes (see Supplementary Figs. 2–5 for full results). (**A**) Beta-band PC; (**B**) Occurrence rate of CAP4; (**C**) Mean lifetime of CAPs 2, 4, and 7; (**D**) Occurrence rates of long-range transitions involving two polarized CAPs, i.e., CAP6 → CAP6 and CAP7 → CAP7; (**E**) Numeric counts of other CAPs visited during CAP6 → CAP6 and CAP7 → CAP7. The lines denote the linear regression models (dashed line indicates a *p* value close to 0.05), whose *p* values are listed in the panels (bold italic font: survived with either FDR or Bonferroni correction).

As our third dataset only contained female participants, we re-calculated all static and dynamic measures on female-only data (35 participants, age: 35.7 ± 16.9 years, range: 6–65 years) using the same analysis procedures to evaluate potential gender bias in the above results^17^. Overall, most age-related changes detected in the all-participant dataset were also detected in this subset of female-only data, illustrated in Fig. 8 and Table 1 (see full results in Supplemental Figs. 2–5).

With the static spectral power measures, the significant beta power increase as a function of age is similarly detected with a whole-brain pattern using the PC metric (Fig. 8A), as well as in the ROI-based regional power measure, in female-only data as compared to the results of all-participant data. On the contrary, the alpha band power only shows regional (not whole brain) age-related effects with a significant increasing pattern using the ROI-based power measure (*p* < 0.05, FDR correction) and an increasing pattern using the PC measure (*p* < 0.05, not survived with the Bonferroni correction) from female-only data, which in general indicate a similar trend of age-related changes in the same direction as the findings from all-participant data. However, no significant changes were detected at the delta and theta bands in female-only data.

Regarding the dynamic CAP measures, the CAPs from female-only data reproduced almost all spatial and temporal characteristics observed in the all-participant data, which included bilateral symmetric spatial patterns in all individual CAPs, anti-correlated CAP pairs, three groups of CAPs defined based on their activation levels (the global high-activation group (Table 1 and Supplemental Fig. 2A): CAPs 2, 4, and 7; the global low-activation group: CAPs 3, 5, and 6; the non-global activation group: CAPs 1 and 8), two polarized states (i.e., CAPs 6 and 7), the 3D distance plot, and low occurrence rates and long lifetimes for the polarized CAPs (Supplemental Fig. 3A). Furthermore, CAPs from the female-only data and all-participant data were one-to-one matched (with only one exception) based on the metric of spatial correlation (Supplemental Fig. 2B). Regarding the age-related changes in the CAP measures, similar patterns have been detected in female-only data compared with all-participant data (Table 1 and Fig. 8). As age increases, significantly increased occurrence rates and significantly decreased lifetimes were observed in the global high-activation CAPs (*p* < 0.05, Bonferroni correction, Fig. 8B,C). Slightly different from the all-participant data, the CAP of significant occurrence changes was not the one with the global highest activation (i.e., CAP7) but the one with the global second-highest-activation CAP (i.e., CAP4). However, CAP7 did show age-related increasing occurrences although it was not survived with the Bonferroni correction (Supplemental Fig. 3B). Similar as for all data (Fig. 4), as age increased, the occurrence rates of the long-range transitions involving the polarized CAPs (i.e., CAP6 → CAP6 and CAP7 → CAP7) also increased (*p* values close to 0.05, without correction, Fig. 8D). Meanwhile, the robust age-related effects of statistical significance on the measure of the numeric counts of other CAPs visited during CAP6 → CAP6 and CAP7 → CAP7 (*p* < 0.05, Bonferroni correction, Fig. 8E) were reproduced. The values of this dynamic measure showed different directions of age-related changes (decrease in CAP6 → CAP6 and increase in CAP7 → CAP7), which were consistent with the directions of changes from all-participant data (see Fig. 4C). It is also noted that no extra age-related changes of statistical significance were identified in female-only data as compared to all-participant data.

In addition, several similar relationships between static spectral power measures and dynamic CAP measures reported above have been identified in female-only data of statistical significance (*p* < 0.05, FDR correction, Supplemental Fig. 5). Specifically, the beta band PC score was positively correlated with the occurrence rate of CAP2 (*p* < 0.05, with FDR correction), negatively correlated to the lifetime of the two global high-activation CAPs (i.e., CAPs 2 and 4) (*p* < 0.05, with FDR correction), positively correlated to the occurrence rate of CAP7 → CAP7 (*p* < 0.05, with FDR correction), and negatively correlated to the occurrence rate of CAP6 → CAP6 (*p* < 0.05, not survived with correction).

## Discussion

In the present study, we used resting-state EEG to investigate age-related changes in dynamic whole-brain patterns as whole-brain analysis has been suggested fundamental in understanding how age affects integration of information in the human brain^25^. We characterized whole-brain electrical activities on the cortical surface with a set of transient brain states, named as co-activation patterns, and identified multiple age-related changes in the dynamics of these transient brain states. The dynamic measures showing age-related changes included ones for individual CAPs (i.e., occurrence rate and lifetime) and those involving the entire set of identified CAPs (i.e., between-CAP transition durations based on number of timeframes and numeric counts of other CAPs visited). These age-related changes detected in dynamic whole-brain measures were further supported by age-related changes observed in the classic static measures of alpha and beta powers in two important aspects: whole-brain patterns and directions of changes. Finally, we reproduced these findings on data of female-only participants.

Our main observation was an increased occurrence rate and/or decreased mean lifetimes of several CAPs of globally high activations, i.e., CAPs 2 and 5 of statistical significance with corrections and CAP4 of statistical significance without correction, but not in the CAPs of global low-activations (i.e., CAPs 1, 3, and 8). Among them, CAP2 represented the brain state with the highest activations at the global scale (e.g., doubled in magnitude as compared with the CAP of the secondary highest activations, CAP4, Fig. 2), while CAPs 4 and 5 represented the brain states of global high activations with specific regional focus, e.g., the precuneus and V1 in CAP5. The highest activation and the lowest occurrence rate of CAP2 characterized itself similar to the brain state identified in fMRI dynamic FC studies that show transient strong interactions among sensory or cognitive networks/regions^3,21,30^. Other CAPs of global high activations seemed to represent intermediate brain states visited during the transition from brain states at the global low activation (i.e., CAPs 1, 3, and 8) to CAP2. The age-related lifetime decreases in these global high-activation CAPs were also consistent with the phenomenon that the older adults spend less time in the above discussed fMRI FC state. It is noted that similar findings have been reported in brain states defined on sensor-level EEG data, i.e., microstates^20,32^, in which age-related occurrences increase and lifetimes decrease are detected in microstates involving high activations with spatial focus on the anterior cingulate cortex^37^.

Secondly, motivated by the structured transition maps among the entire set of CAPs (Fig. 2) and different expressions of age-related changes in individual CAPs of global high and low activations, the present study further investigated the long-range transitions between the CAP of globally lowest activations, i.e., CAP1, and the CAP of globally highest activations, i.e., CAP2, the two polarized brain states in the 3D distance plot. Interestingly, the age-related changes were not observed in long-range transitions between CAPs 1 and 2 (i.e., CAP1 → CAP2 and CAP2 → CAP1), but between the consecutive visits of CAP1 without visiting CAP2 (CAP1 → CAP1) or between the consecutive visits of CAP2 without visiting CAP1 (CAP2 → CAP2). Age-related occurrences increase of CAP2 → CAP2 (Fig. 4A) was detected (*p* < 0.01) even after regressing out the effect from the significantly increased occurrence of CAP2 (Fig. 3) (excluding the effect of occurrence increase of CAP2 on occurrence increase of CAP2 → CAP2). The increased rate of consecutive visits of CAP2 might suggest decreased stability in maintaining the highest-energy brain state and increased fluctuations back and forth between the highest-energy brain state and other global high-energy brain states (see the high spatial similarity between CAP2 and CAP4, cc = 0.73), which can also explain the decreased lifetime of CAP2. Such phenomena are also consistent with the compensation mechanism^38^, which can be used to explain the increased occurrence of the highest-energy brain state. In addition, age-related decreased numeric counts of other CAPs visited during the consecutive visits of CAP1 were observed, while opposite age-related changes (i.e., increased numeric counts of other CAPs visited) during the consecutive visits of CAP2 were identified (Fig. 4C). The results associated with CAP2 provide the plausible evidence that, as age increases, the deviations from the brain state of the highest energy being visited could be increasingly further away, in the numeric counts of brain states, before fluctuating back, which support the theory of reduced stability when visiting the highest-energy brain state. On the other hand, the results associated with CAP1 might suggest an independent phenomenon about whole-brain age-related changes where human brains are more trapped in states of global low activations as age increases. Considering the relatively high occurrence of global low-activation brain states (i.e., CAPs 1, 3, and 8, Fig. 3A) and the fact that the occurrence rate of CAP1 → CAP1 is about 20 times more than the occurrence rate of CAP2 → CAP2 (4.2% vs. 0.2%, Fig. 4A), these global low-activation brain states might represent the baseline brain states under resting conditions and correspond to the fMRI FC state that has been reported with longer time spent in older adults and characterized by weak interactions throughout the whole brain^3,21,30^. However, the increased challenge of exiting low-energy brain states has not been transformed into reduced occurrences of CAP1 → CAP2 in the present study, which might be due to the fact that all participants in the present study were healthy individuals.

In summary, the results indicate that the whole-brain dynamics in resting human brains are largely maintained at the low-energy states, but transiently deviate from these baseline states towards the high-energy state showing co-activation patterns (i.e., CAPs). However, the age-related effects expressed among CAPs are more associated with the dynamics of the high-energy states than the low-energy state (only one measure of statistical significance). These age-related effects in general suggest reduced stabilities (shorter lifetime) and more fluctuations (more visits) when visiting the high-energy brain state, as well as potential compensation mechanisms^38^. The identified low- and high-activation brain states might correspond to fMRI-derived dynamic FC states of weak or strong interactions throughout the brain^30^, respectively, which needs to be clarified with future studies. Furthermore, reduced stabilities suggest plausible increased complexity of brain activations, which have been largely reported in both fMRI and EEG/MEG studies^17,26^. Since the concept of CAPs in EEG is relatively new^39^, the functional interpretations of these age-related effects might require further assessment and be consulted towards CAP studies in fMRI^34,40,41^. At the same time, the present study further indicates these whole-brain dynamic age-related effects are supported by whole-brain age-related changes in classically defined alpha and beta powers as discussed below.

Among all age-related spectral power measures investigated in the present study, the most reliable ones are the alpha and beta band powers. Both alpha and beta powers show significantly increased patterns as age increases and the spatial pattern of whole-brain coverage are duplicated in both ROI-based (Fig. 5) and PCA-based analyses (Fig. 6). The whole-brain spatial pattern of these changes is consistent with the whole-brain pattern of those age-related effects observed in CAPs. Moreover, the directions of these band power changes, i.e., increasing as age increases, are consistent with increased occurrence rates of CAPs of global high activations (i.e., more energies). It is noted that regional (not whole-brain) age-related power changes are also observed in the delta and theta bands (Fig. 5). In particular, the regions showing age-related delta power changes, i.e., precuneus and V1, correspond to the regional focuses in CAP5 on the background of relatively weak global high activations. The direction of this delta power change is consistent with decreased lifetime of CAP5 (i.e., less energies). The relationship between the age-related whole-brain dynamic CAP measures and the classical alpha and beta powers are further supported by their significant correlations (Fig. 7). These facts indicate that age-related effects observed in both CAPs and spectral powers may reflect the same underlying sources. Moreover, more pairs (four out of five) showed significant correlations with age-related CAP measures in the beta band than the alpha band, which indicated that beta band power might be a more reliable biomarker for age effects when spectral powers are considered. This is supported by literature as age-related increases on both regional^2,8,19^ and whole-brain beta powers^8^, have been well reported. On the contrary, age-related alpha power changes have been more complicated in which a quadratic relationship^8^, decreases^2,16,42^, or no changes^23^ have all been reported. This might be due to the slowing of alpha rhythm (therefore the definitions of alpha band might be different at different ages) related to physiological aging^2,8,16,42^.

For an accurate interpretation of the results in the present study, some potential limitations must be considered. First, the present age range is much wider than previous studies that only compared young adults and older adults^17,20^ or focused mainly on maturation^8^. The underlying sources leading to observed age-related changes of whole-brain dynamics might have resulted from complex interplays of multiple physiological and/or neurophysiological events^2^. In particular, while we examined age-related changes in a linear manner, nonlinear changes have been reported^8,10^. This might be due to the facts of relatively less number of samples^17^ (see more discussions below) and missing/insufficient samples on both ends of the lifespan (< 6 and > 65 years old), where an MEG study^8^ (18–89 years old) indicates the peak of a quadratic function of age on neural oscillations around 60 s and changes are monotonic before reaching the peak. Other contributing factors might also include spatial and spectral contents of measures used in examining the age effect as different spatial^10^ and spectral^8^ measures have indicated different patterns on age. Secondly, as a study to examine age-related changes during the lifespan, the number of participants was relatively low in the present study and the age and gender distributions across all samples were not uniform. Regarding gender, we have addressed it by repeating the same analysis on data from female-only participants and were able to replicate similar findings (Fig. 8 and Supplemental Figs. 2–5). Thirdly, EEG data were recorded at different institutions using different EEG systems. Although we aligned them into the same space, i.e., cortical source space, before examining age-related effects to minimize the effects from these differences, it is important to replicate the present analysis on EEG data recorded from more uniform recording protocols. Fourthly, since the EEG data analysis in the present study were based on existing data from previous studies, no consistent data were available for us to examine potential social and culture effects on these identified age-related patterns, while the effects of culture have been recognized in the literature^43^. With EEG data of participants from different culture and social backgrounds available^17^, the same methodology could be used to study such effects in the future. Finally, the present study is limited by its nature of cross-sectional design and its findings need to be verified in a longitudinal design in the same participants.

## Methods

### Datasets

The datasets reported in the present analysis were pooled from three separate studies involving a total of 73 healthy participants (age range: 6–65 years, 35 females). The first study was conducted at the University of Texas Southwestern Medical Center and approved by its local Institutional Review Board. Written informed consent was obtained according to the Declaration of Helsinki for each adult participant and informed parental consent was obtained for individuals younger than 18 years old. As a part of the first study^44^, resting-state high-density EEG data of 5 min with eye closed were collected in each of 19 healthy participants (age: 13 ± 6 years, range: 6–25 years, 6 females) at a sample frequency of 512 Hz using a Biosemi ActiveTwo 128-channel 24-bit resolution system. No sMRI was acquired in this study and age-averaged template MRI data^45^ were used to generate forward models for EEG cortical source imaging. The second study was approved by the Institutional Review Board at the University of Oklahoma Health Science Center (OUHSC) and written informed consent was obtained from all healthy participants. In the second study^46^, resting-state high-density EEG data of 10 min with eyes closed were collected in each of 34 healthy participants (age: 24 ± 5 years, range: 18–38 years, 9 females) at a sample frequency of 1000 Hz using the 128-channel Amps 300 amplifier (Electrical Geodesics Inc., OR, USA). Individual sMRI was acquired via a GE MR750 scanner using GE's "BRAVO" sequence: FOV = 240 mm, axial slices per slab = 180, slice thickness = 1 mm, image matrix = 256 × 256, TR/TE = 8.45/3.24 ms. The third study was conducted at the Laureate Institute for Brain Research, Tulsa, and approved by Western IRB. Written informed consent was obtained from all participants before the start of all procedures. As a part of the third study^47^, resting-state high-density EEG data of 5 min with eye closed were collected in each of 20 healthy participants (age: 49 ± 7 years, range: 30–65 years, 20 females) at a sample frequency of 1000 Hz using a 126-channel BrainAmp amplifier (Brain Products GmbH, Munich, Germany). Individual sMRI data were acquired on a General Electric (GE) Discovery MR750 3 T MRI whole-body scanner (GE Healthcare, Milwaukee WI, USA), which had the parameters: FOV = 240 mm, axial slices per slab = 190, slice thickness = 0.9 mm, image matrix = 256 × 256, TR/TE = 5/2.012 ms, acceleration factor R = 2, flip angle = 8°, inversion time TI = 725 ms, and sampling bandwidth = 31.2 kHz. For the second and third studies, MRI data from individuals were used to generate forward models. EEG sensor positions and three landmark fiducial locations (i.e., nasion, left and right pre-auricular points) were digitized by the Polhemus Patriot system.

### EEG preprocessing

EEG data from three studies were preprocessed using the same procedure at individual participants with the EEGLAB toolbox^48^. First, EEG data was filtered by a band-pass filter of 0.5–100 Hz, and a notch filter of 58–62 Hz. Second, noisy channels and independent components (ICs) of artifacts related to ocular, muscular and cardiac activities, were identified by the *FASTER* plugin^49^ and visual inspection. Third, identified noisy channels were interpolated and artifactual ICs were removed, respectively. Finally, EEG data were down-sampled to 250 Hz and re-referenced to the common average to get the “cleaned” data. It is noted that no EEG segments were rejected to maintain the continuity of data. After preprocessing, EEG data from three studies were unified to have the same spectral band, the same sampling frequency, and the same reference.

### EEG cortical source imaging

Cortical source imaging was performed to reconstruct brain sources over the cortical surface from scalp EEG (Fig. 1A), which deconvoluted the effects of volume conductors on electrical signals and transformed electrical signals from different studies and different individuals into the same spatial domain, i.e., the cortical surface. First, Freesurfer^50^ was used to segment individual sMRI data to extract the surfaces of the scalp, skull, and brain for building individual volume conduction models, and the interface between white and gray matters for the cortical current density (CCD) source model. Second, the surfaces of volume conduction model and the CCD model were tessellated into triangular elements (volume conduction model: 10,242 nodes and 20,484 triangles, CCD model: 20,484 nodes and 40,960 triangles), respectively. For the CCD model, the nodes on the medial wall adjoining the corpus callosum, basal forebrain, and hippocampus, were excluded from the source space, leading the total number of sources as 18,715. The electrical conductivities of the scalp, skull, and brain were assigned as 0.33/Ωm, 0.0165/Ωm, and 0.33/Ωm, respectively. EEG sensor locations were registered on the scalp surface by aligning three landmark fiducial points from both EEG and MRI recordings. Based on these models, the boundary element method^51^ was used to build the forward relationship: **Φ**(*t*) = **L**·**S**(*t*), where **L** is the lead field matrix; **Φ**(*t*) and **S**(*t*) are functions of time for scalp EEGs and dipole current source amplitudes, respectively. The minimum-norm estimate^52^ was used to reconstruct dipole current source amplitudes on the cortical surface: **S**(*t*) = **L**^T^·(**L**·**L**^T^ + *λ*·**I**)^−1^·**Φ**(*t*), leading to the cortical tomography of time-varying current source amplitudes. *λ* was the regularization parameter and selected via the generalized cross validation method^53^ and **I** was the identity matrix. To control the quality of reconstructed cortical sources, the automatically selected *λ* values beyond three standard deviations of all values of each participant were considered as outliers and interpolated with the neighboring ones.

### Whole-brain dynamic measures from CAPs

To obtain whole-brain dynamic metrics, a series of processing steps were performed on cortical tomographic current source data (Fig. 1C)^39^. Briefly, reconstructed cortical tomographic current source data were first down-sampled to 100 Hz and their instantaneous amplitudes were calculated^54,55^. Second, 100 ROIs over the cortex were defined based on an atlas with each ROI representing a parcel of cortical units of functional similarities^56^ and ROI-level envelop data were calculated as average values among all cortical sources within the same ROI. Third, ROI-level envelop data were then converted into z scores (i.e., subtracting the mean and dividing the standard deviation across time per ROI) for each participant and concatenated across participants. Fourth, K-means clustering using the L1-norm distance as the measure was performed on the concatenated data to extract CAPs. K-means clustering was performed with a varied cluster size from 2 to 20, and we finally chose 8 using the metric of explained variances^57^, as well as to achieve the balance of revealing more distinguishable CAPs but producing less duplicated CAPs in terms of their spatial patterns. After obtaining time frames for individual CAPs, their spatial and temporal patterns were extracted accordingly. The whole-brain cortical spatial tomography of each CAP was defined as the averaged map across all time frames (z scores) belonging to the CAP. To investigate the relationship in any pair of CAPs, the spatial correlation coefficients, and L1-norm distances among their cortical tomographies were calculated. To investigate the relationship for the entire set of eight CAPs, we visualized all L1-norm distances in a 3D space using a multidimensional scaling tool from MATLAB (i.e., cmdscale.m). Here, each CAP represented a transient brain state and, therefore, it had temporal measures (Fig. 1C), including occurrence rate, lifetime, and immediate transition among states. An occurrence of a CAP was defined as multiple consecutive time frames that were assigned to the same CAP, and the occurrence rate of a CAP was the ratio between the total number of occurrences of a specific CAP and the total number of occurrences of all CAPs in each participant. Lifetime of a CAP was defined as the number of consecutive time frames in an occurrence of the CAP, and its mean lifetime was first calculated in individual participants, and then averaged over all participants. Immediate transition among states were defined as the transition from one CAP occurrence to the following occurrence of a different CAP. The immediate transition rate was calculated as the number of immediate transitions between any paired CAPs normalized by the total number of immediate transitions in each participant. Beyond immediate transitions, the long-range transition pattern was also investigated between two polarized CAPs, i.e., one with the whole-brain lowest activations and the one with the whole-brain highest activations (CAP1 and CAP2, respectively, in Fig. 2). Specifically, four types of temporally non-overlapped long-range transition were defined, which were two transitions between the same polarized CAP (named as the same-CAP transitions), i.e., CAP1 → CAP1 and CAP2 → CAP2, and two transitions between two different polarized CAPs (the different-CAP transitions), i.e., CAP1 → CAP2 and CAP2 → CAP1. Three measures related to these transitions were calculated in individual participants. The occurrence rate of each long-range transition was defined as the number of their occurrences normalized by the total length of recording data. The mean duration of one type of long-range transition was defined as the averaged time over all same transition. Beyond the number of timeframes used to investigate durations of these transitions, another measure, i.e., numeric counts of other brain states visited (other than brain states coded by CAP1 and CAP2) within each transition, was calculated with the consideration that transitions between brain states might be stepwise over discrete events, rather than continuous waves at fixed speed. These participant-level measures were then statistically compared for group-level analyses.

To obtain age-related changes in these whole-brain dynamic measures, linear regression analysis with age as the independent variable and individual measure values as the dependent variables was performed. Here, statistical significance was determined with the Bonferroni correction method for multiple comparisons, i.e., eight for the measures defined on individual CAPs and four for the measures related to transitions between two polarized CAPs.

### Static measures on band-specific powers

Beyond whole-brain dynamic CAP measures, we also examined static measures of band-specific spectral powers on ROIs (Fig. 1B). First, spectral powers at the delta (1–4 Hz), theta (4–8 Hz), alpha (8–13 Hz), and beta (13–30 Hz) bands were calculated on each cortical source point using *pwelch* function in Matlab (version 2017a). Second, to control for false positive^58^, band-specific power values were calculated as the average values across all source points within a ROI for all 100 ROIs. These power values were log-transformed to normalize their statistical distributions towards the Gaussian distribution before performing parametric statistical analyses. Third, PCA was performed to further reduce the dimensions of ROI-based whole-brain spectral power data into a few dominant principal components (PCs). More precisely, the power values in all ROIs at each frequency band from all participants were decomposed into PCs using the *pca* function in Matlab (version 2017a), respectively. A threshold of 70% for the total variance explained was used as the criterion to select the number of PCs^59^ for further investigations, resulting in only the first PC for all frequency bands.

To probe age-related band-specific power changes, linear regression analysis with the age as the independent variable and log-transformed power values for each ROI or the score (or weight) of the selected PC at each frequency band as the dependent variable was performed. For the regression models on power values among four frequency bands, statistical significance was determined via the FDR correction^36^ for multiple comparisons (i.e., 4 frequency bands × 100 parcellations). For the regression models on the PC scores among four frequency bands, statistical significance was determined via the Bonferroni correction method for multiple comparisons (i.e., 4 frequency bands).

### Examining the relationship between dynamic and static measures showing age-related effects

As several age-related static and dynamic measures were significantly detected (see “Results” section), it was of interest to examine whether they were directly correlated, which might indicate that the age-related effects expressed in these measures were potentially from the same underlying neural mechanisms. Therefore, the pairwise correlation analyses were performed between them. To mitigate the effects of multiple comparisons, only whole-brain band-specific spectral power measures, i.e., PC scores, was used against all whole-brain dynamic measures from CAPs. The statistical significance of calculated correlations was determined via the FDR method for multiple comparisons^36^.

## Supplementary Information

Supplementary Figures.

## Data Availability

The datasets analyzed in this study are available from the corresponding author on reasonable request after approved by institutional authorities.
