# Supplementary material for: Age-related changes of whole-brain dynamics in spontaneous neuronal coactivations
Source: Sci Rep. 2022 Jul 15;12:12140. doi: 10.1038/s41598-022-16125-2 (PMC9287374; doi:10.1038/s41598-022-16125-2)
Supplement: Supplementary file 1 — Supplementary Figures. [file 41598_2022_16125_MOESM1_ESM.docx]

**Age-related changes of whole-brain dynamics in spontaneous neuronal coactivations**

Shou et al.,

**Supplemental Figures**

**
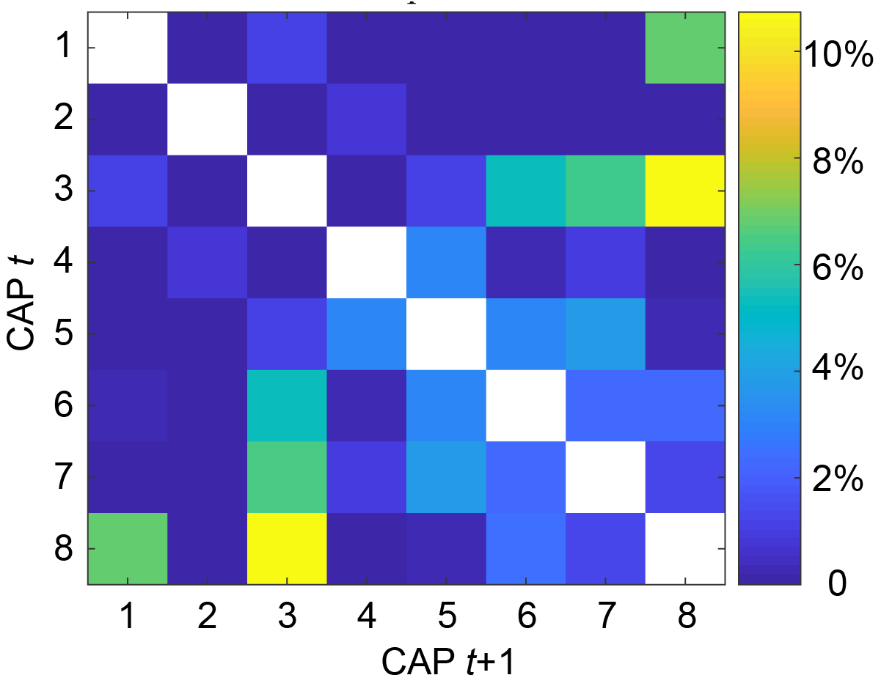
**

**Supplemental figure 1.** The immediate transition probability (one step from time *t* to time *t*+1) among CAPs. The transition probabilities between CAP1 and CAP2 are 1e-6% for CAP1->CAP2 and 0% for CAP2->CAP1. These transition data are used to code the thickness of lines that connect different cCAPs in Figure 2.


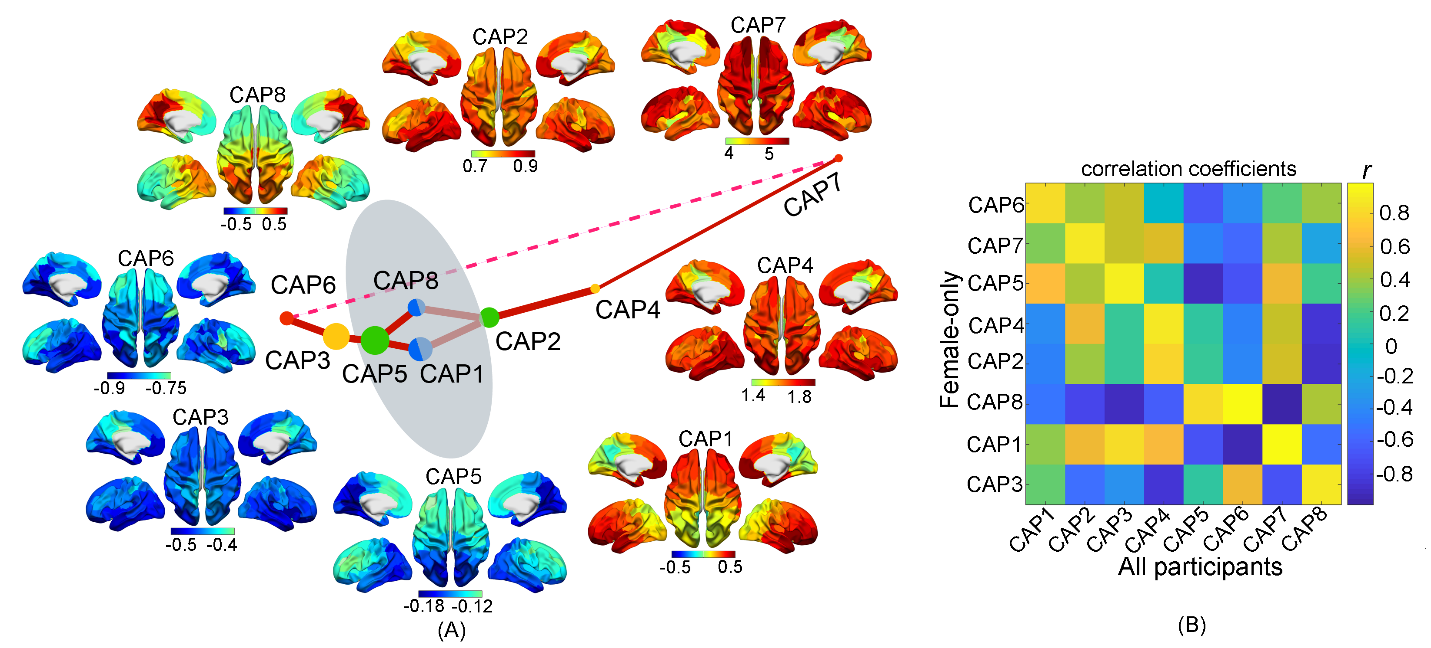


**Supplemental figure 2.** **Reproduced results from data of female-only participants:** (A) Spatial maps (z-score values) of the set of eight CAPs with spatially structured brain-wide patterns and the distance map (via L1-norm distance) between these CAPs projected onto a 3D space. Same figure legends as Fig. 2. (B) Spatial correlation coefficients of matched CAPs between data of all participants and of female-only participants. It is noted that seven (out of total eight) matched CAPs have the largest correlation values, i.e., diagonal elements.


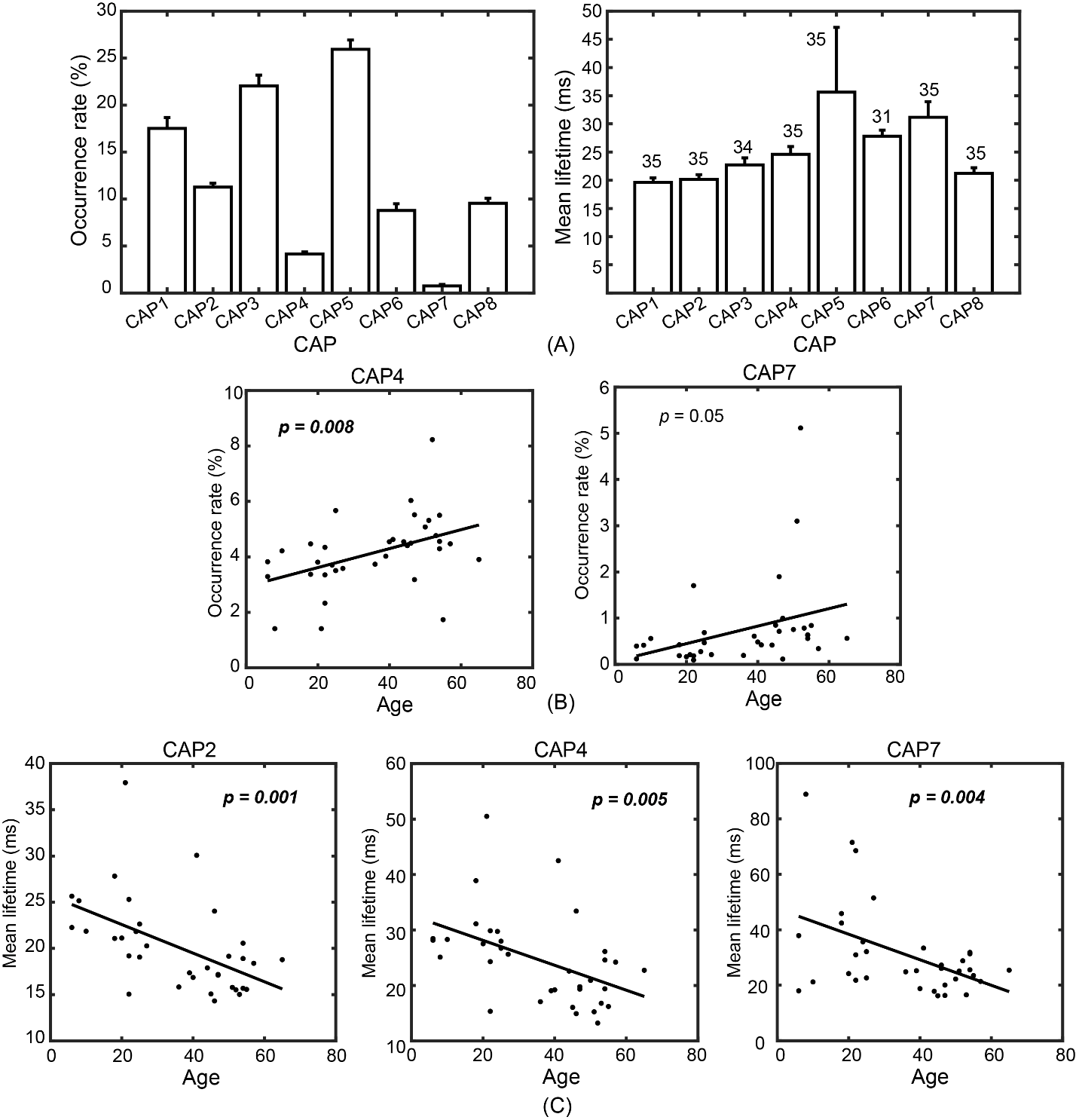


**Supplemental figure 3**. Reproduced CAP-based temporal measures and their age-related patterns identified from data of female-only participants: (A) CAP occurrence rates and mean lifetimes. Numbers on each bar in mean lifetime indicate the number of participants (the total is 35) detected for a corresponding CAP. (B) Age-related occurrence rate changes of CAP4 and CAP7. (C) Age-related mean lifetime changes of CAPs 2, 4, and 7. The lines in (B) and (C) are the linear regression models, whose *p* values are listed in the panels (bold italic fonts: survived with Bonferroni correction).

**
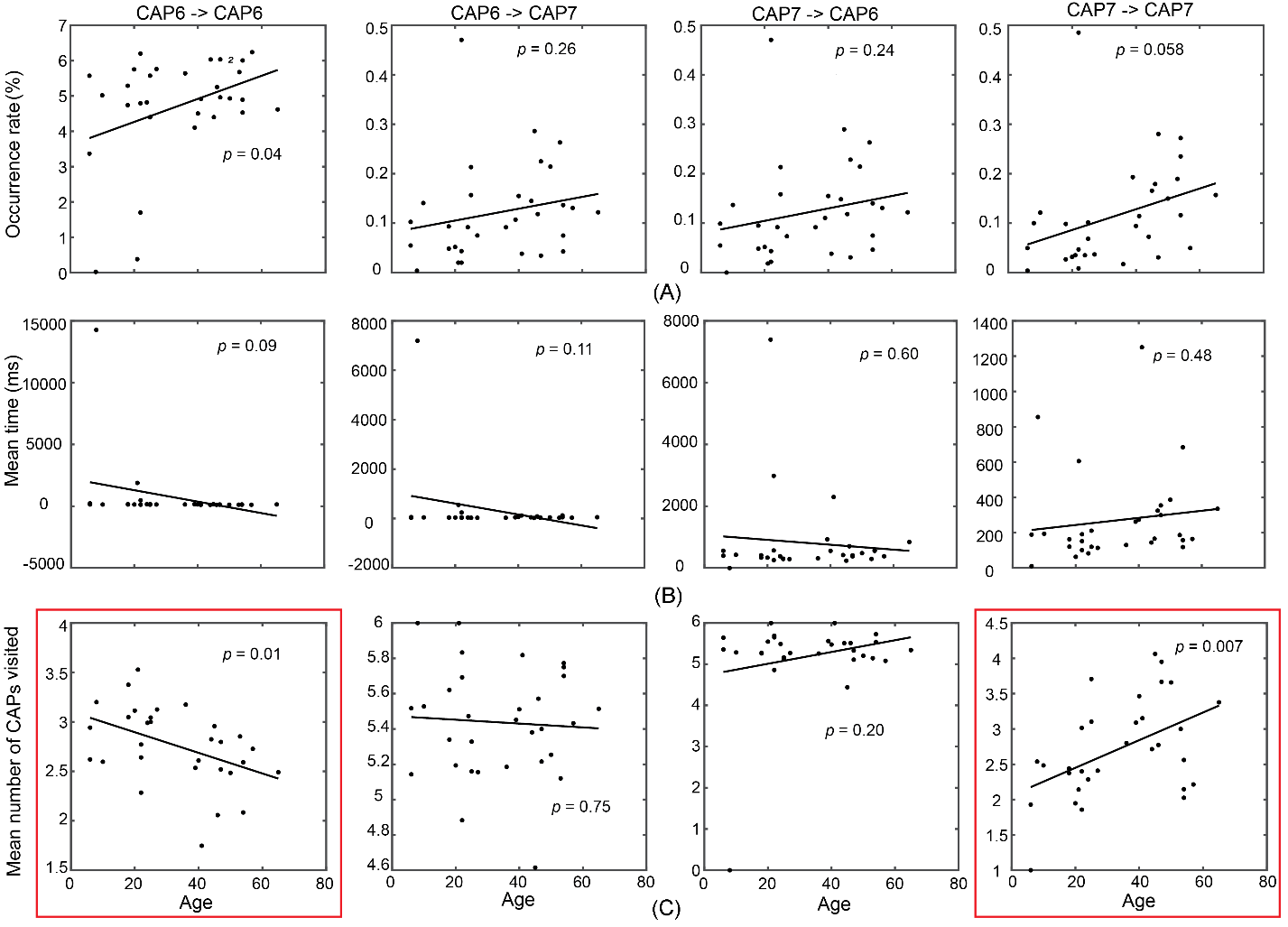
**

**Supplemental figure 4**. Reproduced age-related changes on measures for long-range transitions involving two polarized CAPs from data of female-only participants. (A) Occurrence rate, (B) Mean duration time, (C) Mean numeric counts of CAPs visited. The line in each panel denotes the liner regression model, whose *p* values are listed in the panels (red frames: survived with Bonferroni correction).

**
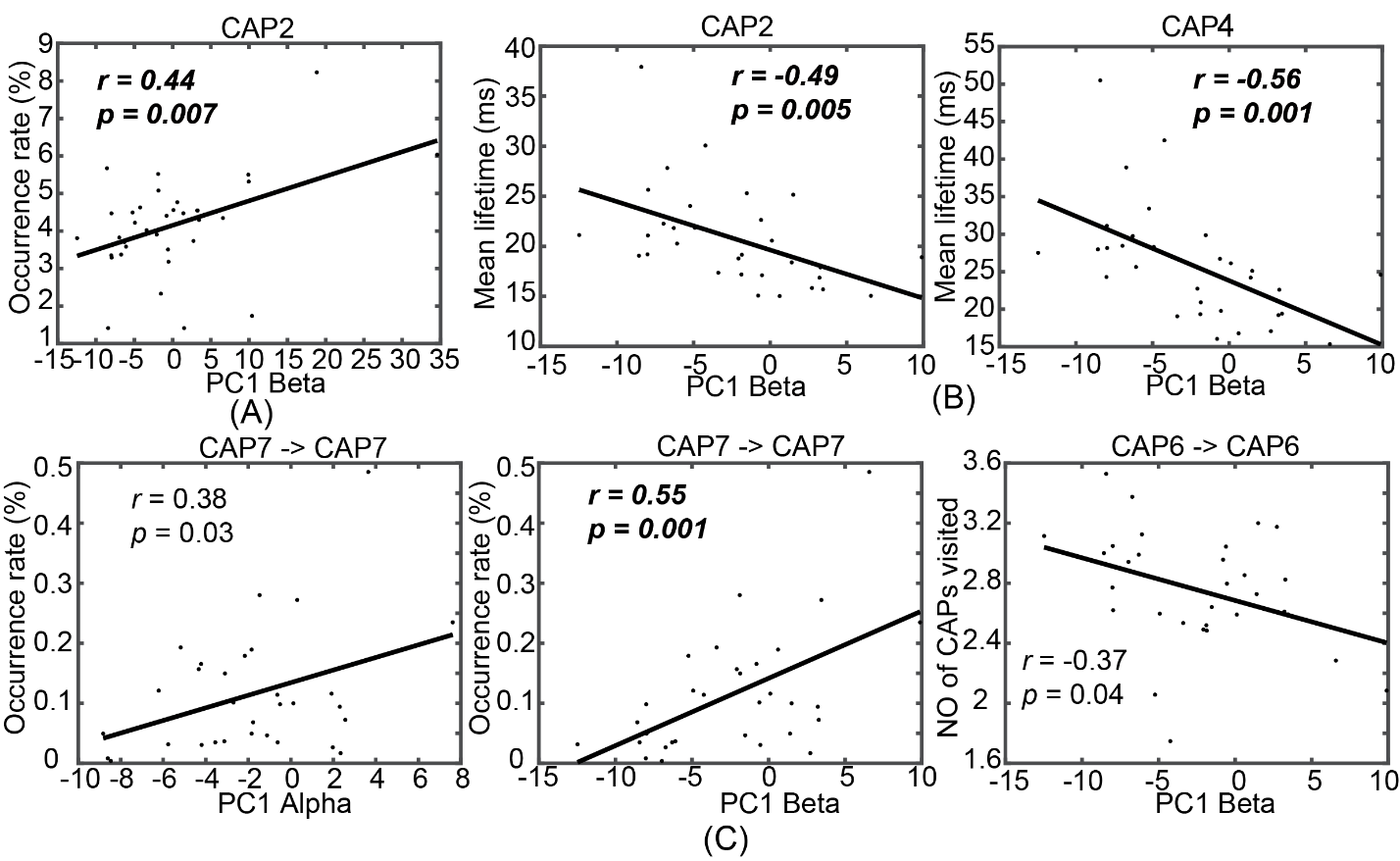
**

**Supplemental figure 5**. Reproduced significant correlations between age-related PCA-based static power measures and CAP-based dynamic measures (*p*<0.01, FDR corrected) from data of female-only participants: (A) Alpha-band PC score vs. CAP 2 occurrence rate, (B) Beta-band PC vs. CAPs 2 and 4 mean lifetime, (C) Alpha- and beta-band PC scores vs. CAP7->CAP7 occurrence rates and numeric counts of other CAPs visited in CAP6->CAP6. The lines denote the liner regression models, while *p* and *r* values obtained from correlational analysis are listed in the panels (bold italic fonts: survived with FDR correction).
